# The role of FAM111B in the malignant progression and molecular regulation of human glioma through the PI3K/Akt pathway

**DOI:** 10.1186/s41016-025-00395-6

**Published:** 2025-05-19

**Authors:** Heng Wang, Junrou Zhu, Haiyang Wang, Wenhao Zheng, Linjie Wang, Jinhao Zhu, Zheng Wang, Quan Du

**Affiliations:** 1 The Fourth School of Clinical Medicine, Zhejiang Chinese Medical University, Hangzhou, China; 2 Department of Neurosurgery, Affiliated Hangzhou First People’s Hospital, School of Medicine, Westlake University, Hangzhou, China

## Abstract

**Background:**

Gliomas represent the most prevalent primary neoplasm in the adult central nervous system. Despite advancements in therapeutic modalities, such as surgical intervention, radiotherapy, chemotherapy, and tumor treatment, the 5-year survival rate of glioma patients remains low. Therefore, there is an urgent need to develop additional treatment methods. Recent studies have suggested that FAM111B is involved in DNA repair, cell cycle regulation, and apoptosis. FAM111B mutations and overexpression are related to cancer.

**Methods:**

We found that FAM111B was significantly overexpressed in glioma tissues compared to the adjacent tissues by analyzing data from the TCGA_GBM&LGG and CGGA databases. Moreover, overexpression of FAM111B was associated with shorter overall survival, and disease-specific survival and tended to increase with disease stage progression. Cellular experiments confirmed these results. These results suggest that overexpression of FAM111B promotes the proliferation, migration, and invasion of glioma cells, whereas the knockdown of FAM111B inhibits these activities. We also found that FAM111B regulated glioma cell proliferation, migration, and invasion via the PI3K/AKT pathway.

**Results:**

FAM111B is capable of enhancing the proliferation, invasion, and migration capabilities of glioma cells and promotes the malignant progression of glioma via the PI3K/Akt signaling pathway.

**Conclusions:**

This is the first study to demonstrate that FAM111B plays a crucial role in the proliferation, migration, and invasion of glioma cells. The malignant phenotype of FAM111B has also been shown to be closely associated with the PI3K/AKT pathway. FAM111B may be a predictive biomarker and a potential therapeutic target for gliomas.

## Background

Glioma is the most common primary neoplasm of the central nervous system in adults and is the most aggressive and fatal manifestation [1]. Despite the implementation of a comprehensive range of treatment modalities, including surgery, radiotherapy, chemotherapy, and tumor treatment, the 5-year survival rate of patients with glioma remains dismally low. This unfavorable prognosis is primarily attributed to the tumor’s invasive characteristics and intrinsic resistance to existing therapeutic approaches [2, 3]. Consequently, there is an urgent need to explore effective treatment strategies. Recently, considerable advancements have been made in molecular-targeted therapies for gliomas. Thus, the identification and mechanistic dissection of key molecules involved in glioma progression are of paramount importance.

The *Homo sapiens* family with sequence similarity 111, member B (FAM111B) is located on chromosome 11q12.1 and encodes a protein that harbors a C-terminal serine/cysteine peptidase-like domain [4–8]. Emerging research has indicated that FAM111B is involved in crucial biological processes, including cell cycle regulation, apoptosis, and DNA repair [9–12]. Moreover, mutations and elevated expression levels of FAM111B have been linked to various fibrotic conditions and malignancies, such as pulmonary fibrosis, thyroid cancer, and lung adenocarcinoma [13–17]. Kawasaki et al. demonstrated that FAM111B modulates p16 levels by directly interacting with and degrading this molecule, thereby augmenting cyclin D1-CDK4 activity in lung adenocarcinoma. This action facilitates cell cycle progression and cellular proliferation, positioning FAM111B as a potential prognostic marker for lung adenocarcinoma [16]. In hepatocellular carcinoma, FAM111B elevates TACC3 expression, thereby activating the PI3K/AKT signaling pathway. PRIM2 enhances FAM111B expression in pancreatic ductal adenocarcinoma, which in turn promotes tumor cell proliferation and migration. This process is accomplished by modulating the PI3K/AKT pathway and facilitating the epithelial-mesenchymal transition [18, 19]. This study aimed to investigate the previously unknown role and molecular dynamics of FAM111B in gliomas. The objective of this study was to clarify how FAM111B contributes to the aggressive characteristics of gliomas and to uncover the specific molecular pathways involved. Our findings revealed that FAM111B affected glioma malignancy by modulating the PI3K/AKT pathway, thereby presenting a new potential avenue for therapeutic intervention in the treatment of glioma.

## Methods

### Data source

RNA sequencing (RNA-seq) data from The Cancer Genome Atlas (TCGA) and the Genotype-Tissue Expression (GTEx) project were obtained from the UCSC XENA database (https://xenabrowser.net/datapages/). This database contains data on the corresponding normal tissues from TCGA and GTEx, along with relevant clinical information pertaining to glioma samples. Additionally, mRNA sequencing data for the Chinese Glioma Genome Atlas (CGGA) dataset were retrieved from the CGGA database (http://www.cgga.org.cn/).

### Tissue sample

Tumor and adjacent normal tissue samples were collected from four patients with glioma at the First People’s Hospital of Hangzhou. This study was approved by the Ethics Committee of the hospital and aligned with the principles outlined in the Declaration of Helsinki. Written informed consent was obtained from all participants prior to their participation, authorizing the use of their biological materials for scientific analysis.

### Cell culture and transfection

Normal human astrocytes (NHAs) and human glioma lines A172, U251, U87, and SNB19 were purchased from Servicebio. These cell cultures were nurtured in DMEM/F-12 solution enriched with 10% fetal bovine serum (FBS) and incubated at a stable environment of 37 °C with a 5% CO_2_ atmosphere. Specific small interfering RNAs (siRNAs) targeting *FAM111B* were acquired from Servicebio. These siRNAs were successfully introduced into the SNB19 and U251 cells using Lipofectamine 2000.

### Western blot assay (WB)

Proteins were extracted from both cell and tissue samples using the RIPA buffer, and their concentrations were determined using the BCA protein assay method. Following extraction, the proteins were separated by 10% SDS-PAGE and transferred onto a polyvinylidene fluoride membrane. This membrane was blocked with 5% non-fat milk for 1 h, then incubated with primary antibodies at 4 °C overnight. The primary antibodies, targeting FAM111B, PI3K, p-PI3K, AKT, p-AKT, and GAPDH, were all procured from Abcam. Post-primary antibody incubation, the membrane was treated with the appropriate secondary anti-rabbit antibodies (Abcam) for 1 h at37°C. Following extensive rinsing in TBST, protein bands were visualized using an ECL chemiluminescence kit. Band quantification was performed using the Quantity One software.

### Quantitative real-time PCR (qRT-PCR)

All primers were synthesized by Sangon Biotech (Shanghai, China). The nucleotide sequences are provided in Table 1.

**Table 1:** Characteristics of patients with glioma based on TCGA

**Primer name**	**Sequence**(5'-3')
FAM111B-F FAM111B-R GAPDH-F GAPDH-R	CGCCAGACAATTCCCAGGATTAG CCAGATTAATAGCATACCGCCTACC GGGAAACTGTGGCGTGAT GAGTGGGTGTCGCTGTTGA

#### RNA extraction

Cells in the logarithmic growth phase were collected. TRIzol reagent (1 mL per 1 × 10⁷ cells) was added and homogenized by pipetting, followed by incubation at room temperature for 5 min. The mixture was centrifuged at 12,000 rpm and 4 °C for 15 min. After visible liquid phase separation, the upper aqueous phase was transferred, and an equal volume of isopropanol was added. The mixture was inverted and allowed to stand, then centrifuged under identical conditions. The RNA pellet was washed with 75% ethanol, centrifuged again under the same conditions, and finally resuspended in DEPC-treated water.

#### RNA quantification

A spectrophotometer was zero-calibrated, and 1 μL of RNA sample was loaded. RNA concentration was measured and recorded.

#### Reverse transcription to cDNA

The reverse transcription system was prepared according to the manufacturer’s protocol of the reverse transcription kit. Reactions were performed sequentially under the temperature and time parameters specified in the protocol. Synthesized cDNA was stored at − 20 °C.

#### PCR amplification

The amplification system was prepared following the instructions by Beyotime Biotechnology. Amplification reactions were conducted with programmed thermal cycling parameters (temperature, duration, and cycle number) as specified in the protocol.

#### Data analysis

Gene expression levels were calculated based on Ct values obtained after reaction termination.

### Colony formation assay

Transfected U251 and SNB19 cells were seeded in 6-well plates at a density of approximately 800 cells/well. The cells were cultured at 37 °C under 5% CO_2_ for 2–3 weeks. After culturing, the cells were fixed with 10% formaldehyde for 20 min, followed by staining with 0.1% crystal violet for 10 min. Cell colonies were quantified using ImageJ software.

### Transwell assay

A low-concentration BD Matrigel basement membrane matrix was used for cell invasion assays. Once the matrix gel solidified, cells that had been cultured without serum for 12 h were placed in the upper compartment of a Transwell setup at a seeding density of 1 × 10^6^ cells/mL. The lower compartment was filled with DMEM/F-12 supplemented with 10% FBS. After a 24–28-h incubation period, the cells that had penetrated through the matrix were fixed using 10% formaldehyde for 20 min and then stained with 0.1% crystal violet for another 10 min. The invading cells were assessed using the ImageJ software for quantitative analysis.

### Wound healing assay

For the scratch assay, the cells were cultured in 6-well plates until 100% confluence was achieved. A pipette tip was used to generate scratches across cell monolayers. Photographic documentation of the wound area was conducted immediately (0 h) and 36 h post-scratch. The extent of wound closure was assessed by measuring the remaining open area using ImageJ software.

### Subcutaneous tumorigenesis experiment in nude mice

Logarithmic cells were taken, and their cell suspensions were prepared at a density of 1 × 10^7^/mL. Using the right hand, a syringe was subcutaneously inserted into the posterior axillary part of the immobilized nude mouse and each nude mouse was inoculated with 100 μL of cell suspension. For the outcome statistics, the longest and shortest diameters of tumors were measured with vernier calipers and the volume was calculated. The nude mice were sacrificed after anesthesia using 4% chloral hydrate, and the subcutaneous xenografts were peeled off, photographed, and weighed.

### Statistical analysis

All presented data are expressed as the mean ± standard deviation (SD) derived from three independent experiments. Data analyses were executed utilizing GraphPad Prism 8.0 software. Survival rates were evaluated using the Kaplan–Meier method. Statistical significance was set at *P* < 0.05.

## Results

### FAM111B expression tends to be positively correlated with glioma malignant degree

**Fig. 1:**
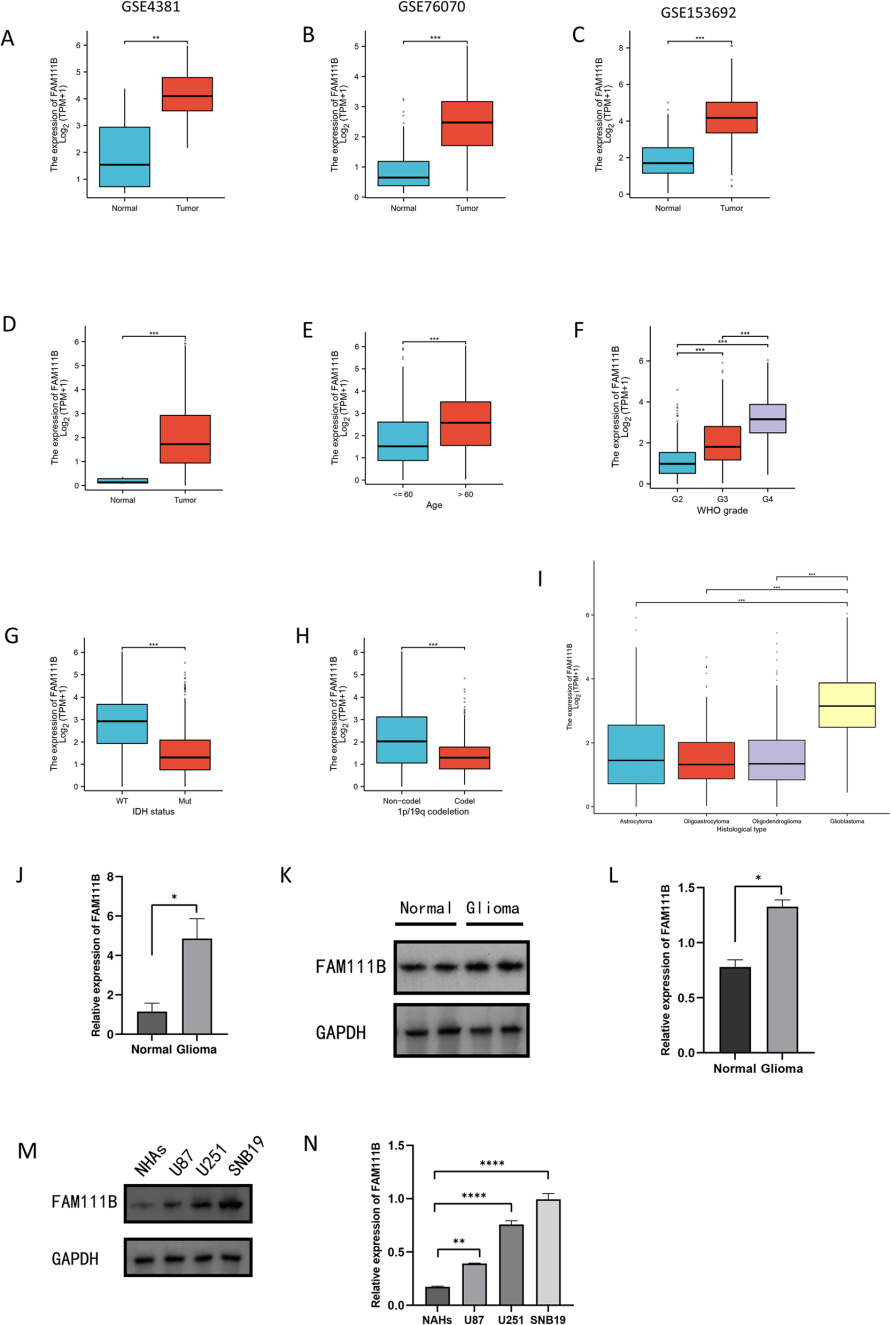
FAM111B was significantly overexpressed in glioma. **A** GSE4381 database showing the expression of FAM111B in glioma and normal tissues. **B** GSE76070 database showing the expression of FAM111B in glioma and normal tissues. **C** GSE153692 database showed the expression of FAM111B in glioma tissues and normal tissues. **D** TCGA database showing the expression of FAM111B in glioma and normal tissues. **E** TCGA database showing the expression of FAM111B in patients with and without senility. **F** TCGA database showing the expression of FAM111B in different WHO grades. **G** TCGA and CGGA databases showing that the expression of FAM111B exhibited the expression of FAM111B in IDH status. **H** The CGGA database showing the expression of FAM111B in 1p/19q codeletion and 1p/19q non-codeletion. **I** The CGGA database showing the expression of FAM111B in different pathological types. **J**, **K**, **L** Western blot and q-PCR showing the expression of FAM111B in glioma and normal tissues. **M**, **N** Western blot showing the expression of FAM111B in glioma and normal cell lines. According to the mean and standard deviation *P < 0.05, **P < 0.01, ***P < 0.001, and ****P < 0.0001. ns, not significant

We examined FAM111B expression in samples from patients with glioma and healthy controls using TCGA_GBM&LGG and CGGA databases, as well as the GSE4381, GSE76070, and GSE153692 datasets. Our findings revealed that FAM111B expression was elevated in glioma tissues compared to that in normal tissues (Fig. 1A–D). Additionally, we explored the association between FAM111B expression and clinical characteristics of gliomas. Known prognostic factors, such as age (specifically, patients older than 60 years), tumor grade (WHO III/IV), isocitrate dehydrogenase (IDH) mutation status, and the absence of 1p/19q codeletion, are associated with glioma malignancy and prognosis. Our results showed that FAM111B expression was elevated in older patients, higher-grade gliomas (WHO III/IV), IDH-mutated gliomas, gliomas without 1p/19q codeletion, and patients with glioblastomas (Fig. 1E–I). These findings suggest that FAM111B overexpression is associated with these clinical parameters, indicating a possible correlation between elevated FAM111B levels, increased malignancy, and poor prognosis in gliomas.

To corroborate our initial findings, we analyzed tumor and adjacent normal tissues from four patients with glioma. WB and qPCR analyses indicated a marked increase in FAM111B mRNA and protein levels in tumor tissues relative to adjacent normal tissues (Fig. 1J–L). Furthermore, we assessed the protein expression of FAM111B in glioma cell lines (U251, U87, and SNB29) and in NHAs (Fig. 1M, N). Consistent with our earlier observations, these glioma cell lines exhibited significant FAM111B overexpression compared to NHAs. These results provide additional support for elevated FAM111B expression in both glioma tissues and cell lines, reinforcing its potential role in glioma pathology.

### FAM111B expression predicts the prognosis of glioma patients and clinical features

**Fig. 2:**
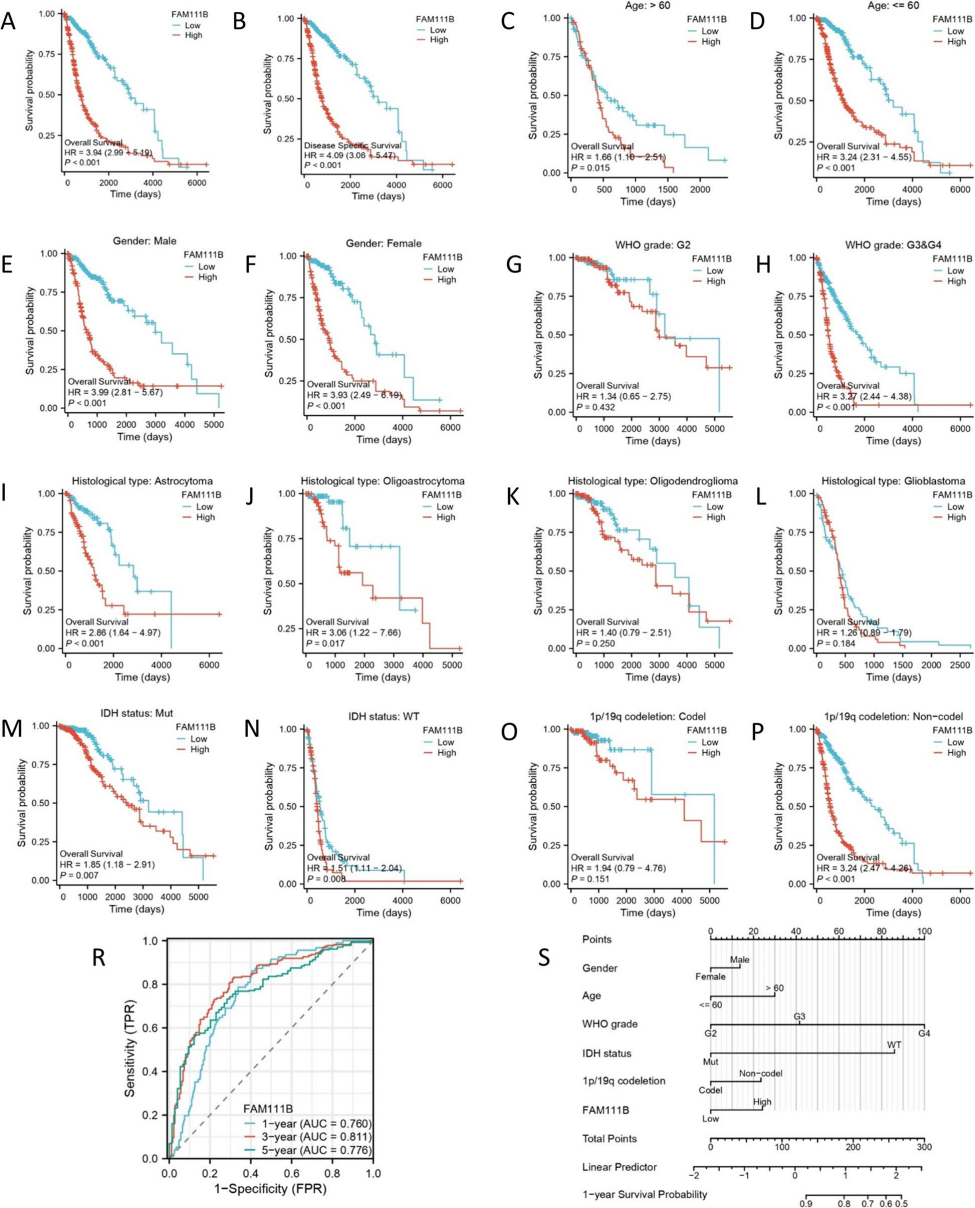
The effect of FAM111B expression on the survival of glioma. **A** FAM111B expression and overall survival in TGGA. **B** FAM111B expression and disease-specific survival in TGGA. **C**, **D** FAM111B expression at different ages in TGGA. **E**, **F** FAM111B expression in different sexes in TGGA. (G, H) FAM111B expression in different WHO grades in TCGA. **I**, **J**, **K**, **L** FAM111B expression in different histological types in TCGA. **M**, **N** FAM111B expression in different IDH statuses in TCGA. **O**, **P** FAM111B expression in different 1p/19q codeletion in TCGA. **R** ROC curves showed that FAM111B was a potential prognostic marker for gliomas based on TCGA. **S** A nomogram was used to predict the 1-year survival probability

To assess the prognostic significance of FAM111B in glioma patients and its association with clinical characteristics, we conducted Kaplan–Meier survival analyses for overall survival (OS) and disease-free survival (DFS) using patient data from the TCGA database. The baseline clinical characteristics of the TCGA cohort are shown in Table 1. Patients with high FAM111B expression showed significantly reduced OS, progression-free (PFS), and DFS (Fig. 2A, B). Furthermore, analyses incorporating both TCGA and CGGA datasets revealed that various clinical parameters, including age (hazards ratio [HR] = 1.66), younger age (HR = 3.24), male sex (HR = 3.99), female sex (HR = 3.93), astrocytoma (HR = 2.86), oligodendroglioma (HR = 3.06), high-grade tumors (WHO III/IV) (HR = 3.27), and 1p/19q non-codeletion status (HR = 3.24), were associated with similar trends in survival probabilities (Fig. 2C–P). However, no significant differences in survival probabilities were observed in patients with low-grade gliomas (WHO II), oligoastrocytomas, glioblastomas, IDH status, and 1p/19q codeletion (Fig. 2G, K–O).

Additionally, through both univariate and multivariate analyses, we established FAM111B as an independent prognostic indicator of gliomas. Univariate analysis indicated significant associations between glioma prognosis and several variables, including FAM111B expression (HR = 2.107), age (HR = 4.668), WHO grade (HR = 18.615), IDH mutation status (HR = 0.117), and 1p/19q non-codeletion status (HR = 4.428) (Table 2). The multivariate analysis corroborated these findings, underscoring the role of high FAM111B expression as an independent risk factor for glioma prognosis. Additionally, the diagnostic potential of FAM111B in glioma was assessed using receiver operating characteristic (ROC) curves. Time-dependent ROC analysis demonstrated that the area under the curve (AUC) values for predicting the 1-year, 3-year, and 5-year survival rates of glioma patients were 0.760, 0.811, and 0.776, respectively (Fig. 2R), highlighting FAM111B’s utility as a reliable biomarker for glioma diagnosis. Furthermore, the calibration curve provided a predictive comparison between the 1-year survival probability and clinical features (Fig. S2). In summary, these findings underscore the significance of FAM111B as a pivotal element in predicting the prognosis and clinical outcomes of patients with gliomas.

**Table 2:** Univariate/multivariate Cox analysis showing that FAM111B can be used as an independent prognostic risk factor for glioma

Characteristics	Total(N)	Univariate analysis	*P* value	Multivariate analysis	*P* value
		Hazard ratio (95% CI)		Hazard ratio (95% CI)	
Age	695				
<=60	552	Reference			
>60	143	4.668 (3.598-6.056)	**<0.001**	1.484 (1.089-2.023)	**0.013**
Gender	695				
Female	297	Reference			
Male	398	1.262 (0.988-1.610)	0.062	1.226 (0.933-1.611)	0.144
WHO grade	634				
G2	223	Reference			
G3	243	2.999 (2.007-4.480)	**<0.001**	1.785 (1.150-2.771)	**0.010**
G4	168	18.615 (12.460-27.812)	**<0.001**	3.874 (2.129-7.047)	**<0.001**
IDH status	685				
WT	246	Reference			
Mut	439	0.117 (0.090-0.152)	**<0.001**	0.276 (0.181-0.421)	**<0.001**
1p/19q codeletion	688				
codel	170	Reference			
noncodel	518	4.428 (2.885-6.799)	**<0.001**	1.246 (0.693-2.237)	0.462
Histological type	695				
Astrocytoma	195	Reference			
Glioblastoma	168	6.791 (4.932-9.352)	**<0.001**		
Oligoastrocytoma	134	0.657 (0.419-1.031)	0.068	1.223 (0.746-2.006)	0.424
Oligodendroglioma	198	0.580 (0.395-0.853)	**0.006**	0.870 (0.528-1.434)	0.586
FAM111B	695	2.107 (1.871-2.373)	**<0.001**	1.310 (1.115-1.539)	**0.001**

### Knockdown and overexpression of FAM111B influence the malignant features of glioma cells

**Fig. 3:**
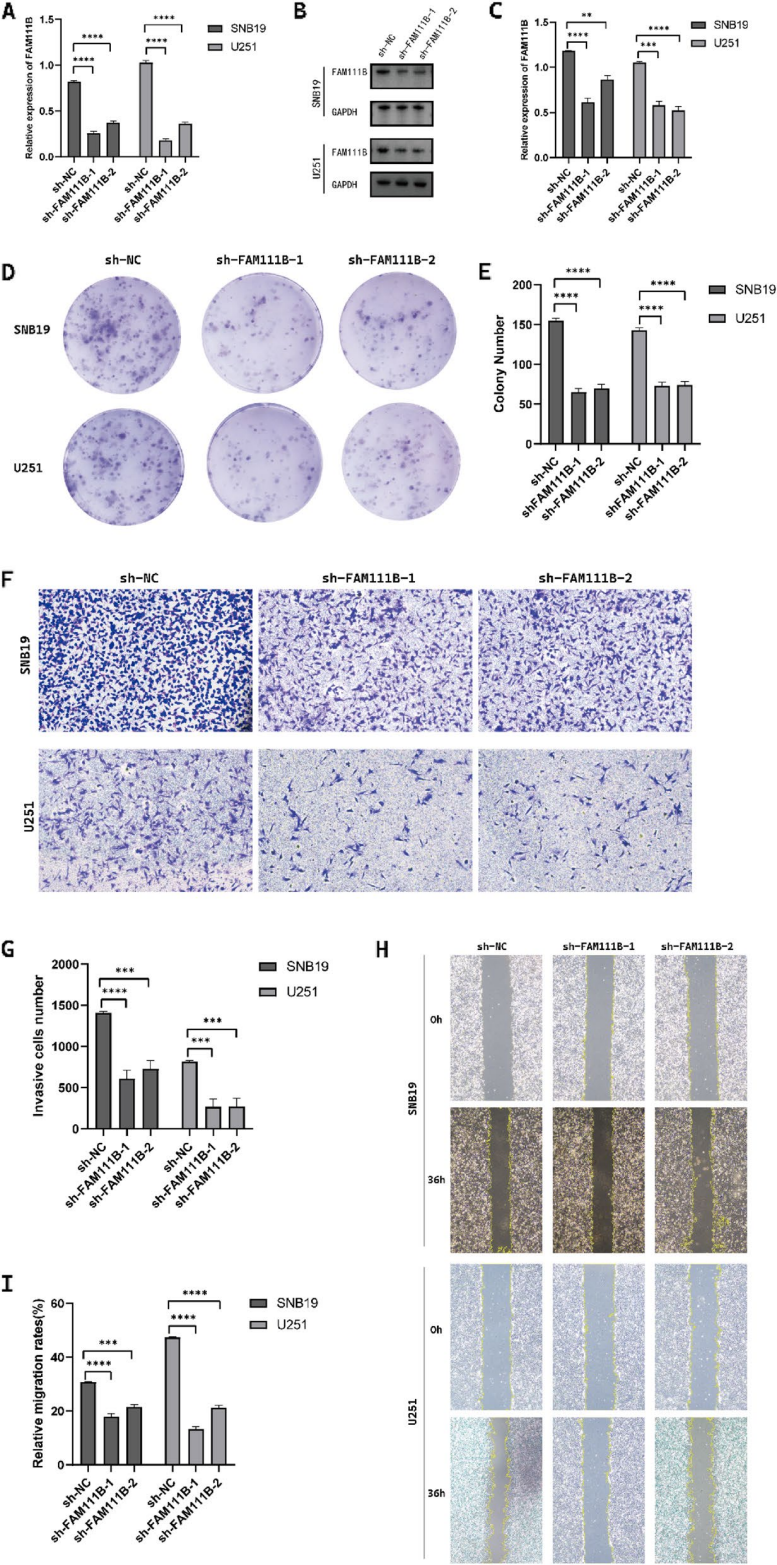
FAM111B knockdown suppressed glioma cell proliferation, migration, and invasion. **A**–**C** Western blot and q-PCR showing the transfection efficiency of FAM111B after transfection with lentivirus in glioma cells. **D**, **E** Colony formation assay showing the colony formation number of SNB19 and U251 cells transfected with lentivirus. Magnification × 1. **F**, **G** Transwell assay showing the number of invasive glioma cells transfected with lentivirus in SNB19 and U251 cells. Magnification × 20. **H**, **I** Wound healing assay showing the amount of open area remaining after transfection with lentivirus in SNB19 and U251 cells. Magnification × 4. According to the mean and standard deviation *P < 0.05, **P < 0.01, ***P < 0.001, and ****P < 0.0001. ns, not significant

**Fig. 4:**
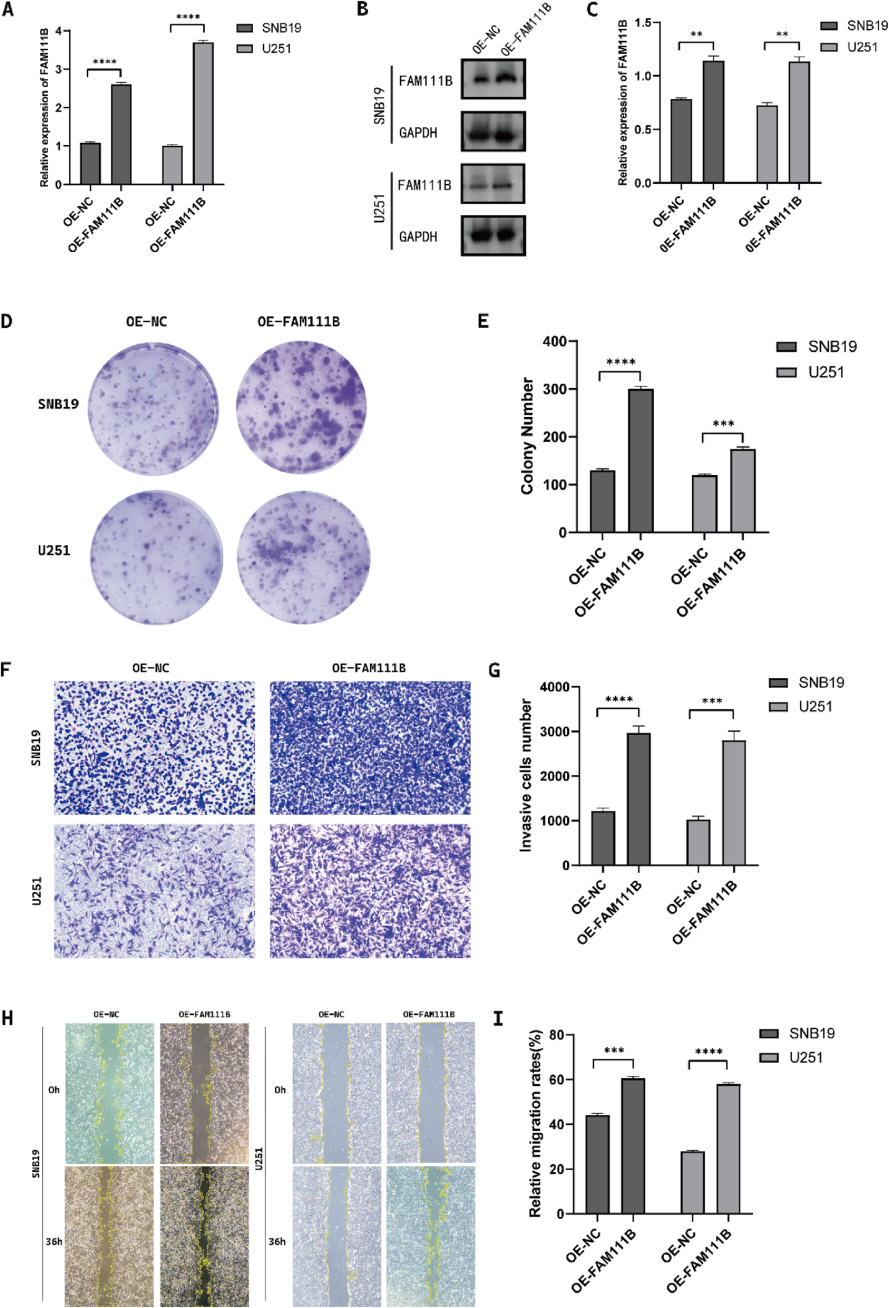
Overexpression of FAM111B promoted the proliferation, migration, and invasion of glioma cells. **A**–**C** Western blot and q-PCR showing the transfection efficiency of FAM111B after transfection with lentivirus in glioma cells. **D**, **E** Colony formation assay showing the proliferation of SNB19 and U251 cells transfected with lentivirus. Magnification × 1. **F**, **G** Transwell assay showing the number of invasive glioma cells transfected with lentivirus in SNB19 and U251 cells. Magnification × 20. **H**, **I** Wound healing assay showing the amount of open area remaining after transfection with lentivirus in SNB19 and U251 cells. Magnification × 4. According to the mean and standard deviation *P < 0.05, **P < 0.01, ***P < 0.001, and ****P < 0.0001. ns, not significant

**Fig. 5:**
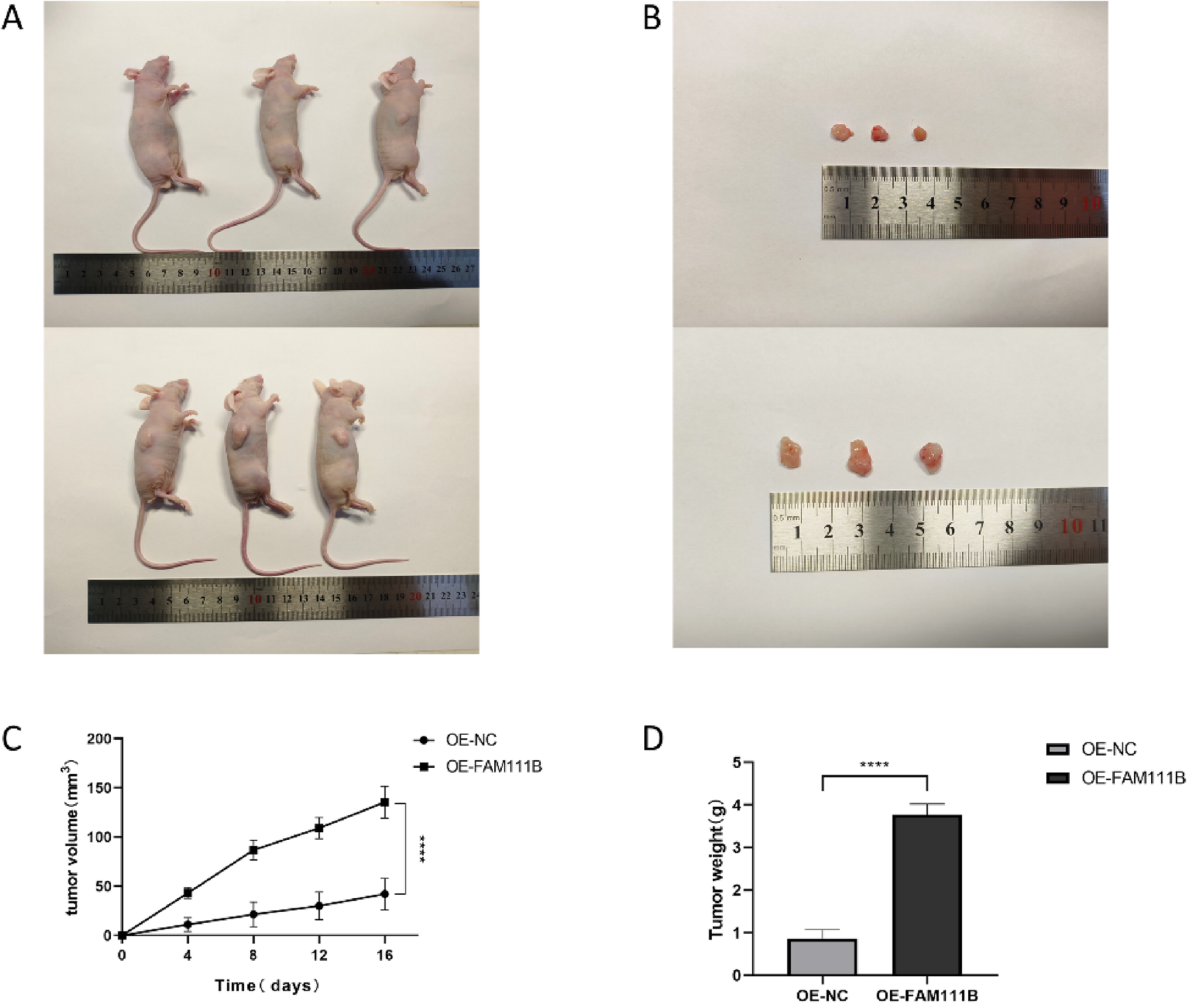
FAM111B promotes glioma cell tumorigenesis in vivo. **A** Nude mice were subcutaneously inoculated with SNB19 cells transfected with FAM111B-overexpressing viral vectors and negative control viral vectors. **B** Subcutaneous tumors in nude mice derived from SNB19 cells transfected with FAM111B-overexpressing viral vectors and negative control viral vectors. **C**, **D** Upregulated FAM111B expression in SNB19 cells of nude mice; subcutaneous xenograft volume and mass significantly increased. According to the mean and standard deviation *P < 0.05, **P < 0.01, ***P < 0.001, and ****P < 0.0001. ns, not significant

To further elucidate the functional roles of FAM111B in gliomas, we employed lentiviral vectors for the overexpression and stable knockdown of FAM111B in glioma cell lines. The transfection efficacy was verified using WB and qPCR. Our data demonstrated that FAM111B expression was significantly decreased in cells transfected with sh-FAM111B constructs (sh-FAM111B cells), whereas it was substantially increased in cells transfected with OE-FAM111B constructs (OE-FAM111B cells), as evidenced by both WB and qPCR analyses (Figs. 3A–C and 4A–C). Colony formation assays revealed that OE-FAM111B cells formed more colonies than the control cells, indicating enhanced proliferation, whereas sh-FAM111B cells showed a marked reduction in colony number (Figs. 3D, E and 4D, E). Furthermore, results from the Transwell invasion assays demonstrated increased invasiveness in OE-FAM111B cells, whereas invasiveness was diminished in sh-FAM111B cells (Figs. 3F, G and 4F, G). Scratch wound healing assays corroborated these findings, showing accelerated closure in OE-FAM111B cells and delayed closure in sh-FAM111B cells after 48 h (Figs. 3H, I and 4H, I). In vivo experiments on subcutaneous tumorigenesis in nude mice were performed to verify the effect of FAM111B expression on the tumorigenic ability of glioma cells. The results showed that the volume and mass of subcutaneous xenografts in nude mice transfected with SNB19 cells overexpressing the FAM111B virus were significantly increased compared to those transfected with the negative control virus, indicating that the upregulation of FAM111B expression significantly promoted the tumorigenesis of glioma cells in vivo (Fig. 5A–D). Collectively, these results suggest that FAM111B overexpression promotes the malignant properties of glioma cells, whereas its knockdown inhibits these oncogenic processes. Consequently, FAM111B appears to be a significant contributor to glioma malignancy.

### *FAM111B regulates glioma cell malignant features *via* the PI3K/AKT pathway*

**Fig. 6:**
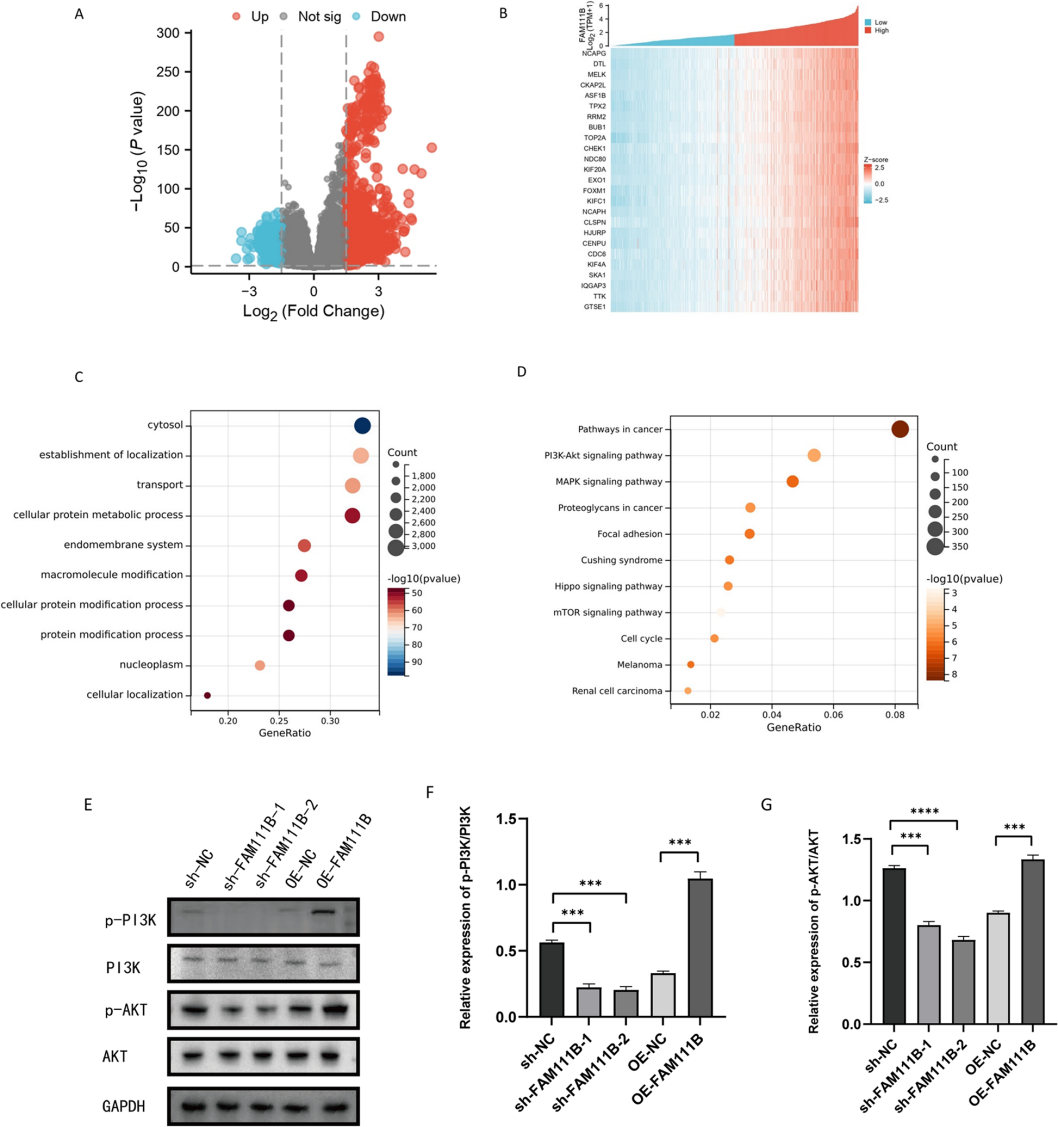
Enrichment analysis of differentially expressed genes in FAM111B. **A** Volcano map of FAM111B-related differentially expressed genes. **B** Heatmap of FAM111B-related differentially expressed genes. **C** Gene Ontology of FAM111B-related differentially expressed genes. **D** The Kyoto Encyclopedia of Genes and Genomes of FAM111B-related differentially expressed genes. **E**–**G** Western blot showing the PI3K/AKT pathway-related protein levels in glioma cells. According to the mean and standard deviation *P < 0.05, **P < 0.01, ***P < 0.001, and ****P < 0.0001. ns, not significant

To investigate the mechanisms by which FAM111B influences glioma biology, we categorized the dataset into two groups based on FAM111B expression: those with expression above (upregulated) or below (downregulated) the median value. We identified differentially expressed genes (DEGs) between these groups, which were visualized using volcano plots and heatmaps (Fig. 6A, B). Using these FAM111B-associated DEGs, we further explored the potential regulatory roles of FAM111B in gliomas by conducting Gene Ontology (GO) and Kyoto Encyclopedia of Genes and Genomes (KEGG) analyses. GO analysis indicated that these genes were involved in a range of biological processes, including cytoplasmic organization, localization establishment, transport, cellular protein metabolic processes, endomembrane system regulation, and macromolecule modification (Fig. 6C). Meanwhile, KEGG pathway analysis revealed that these genes were involved in several key cancer-related signaling pathways, such as the cancer, PI3K-Akt, and MAPK pathways (Fig. 6D).

**Fig. 7:**
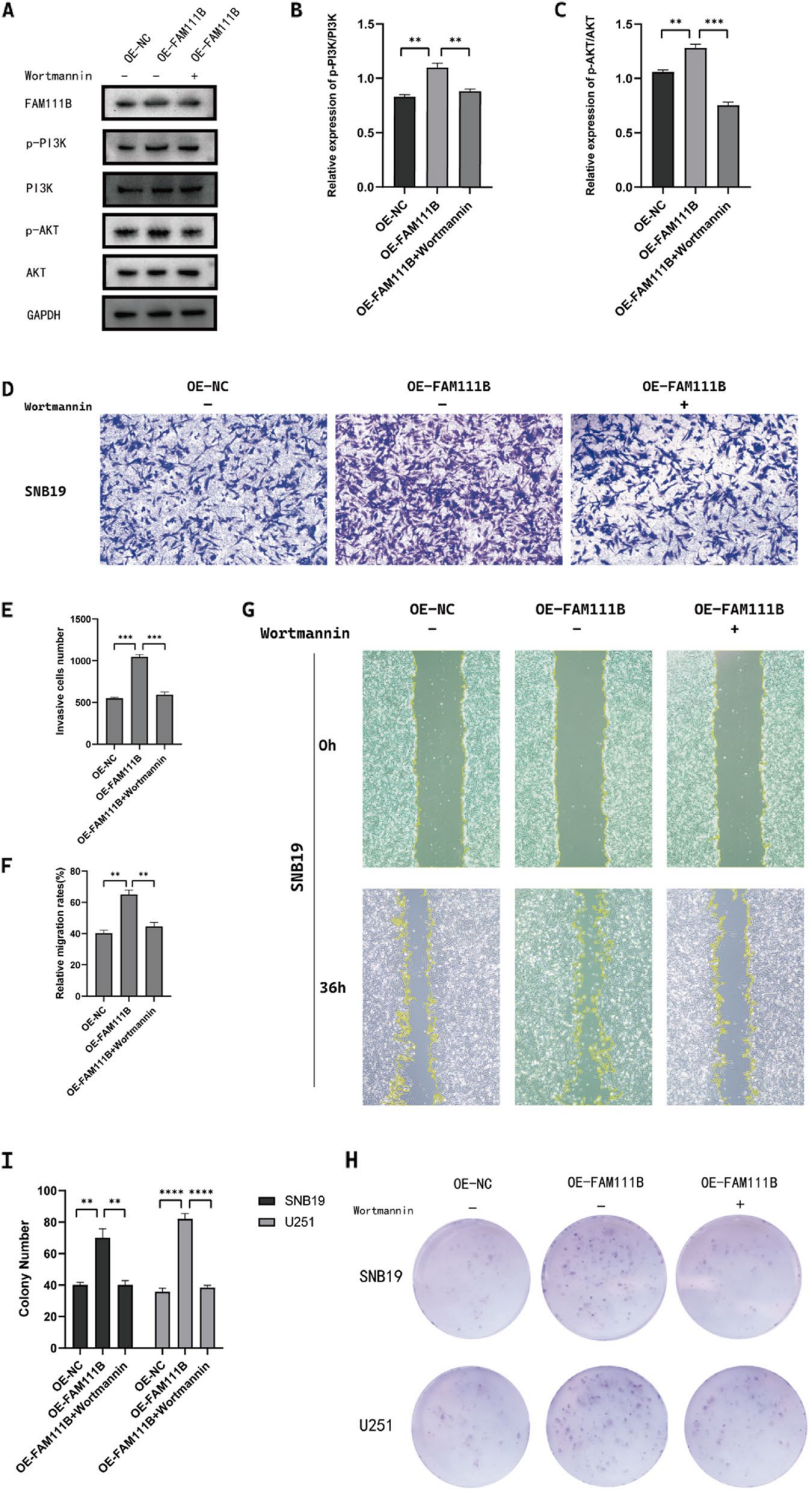
FAM111B regulated glioma cell proliferation, migration, and invasion via the PI3K/AKT pathway. **A**–**C** Western blot showing the PI3K/AKT pathway-related protein levels after using a PI3K inhibitor in SNB19 cells. **D**, **E** Transwell assay showing the number of invasive SNB19 glioma cells after treatment with a PI3K inhibitor. Magnification × 20. **G**, **F** Wound healing assay showing the amount of open area remaining in SNB19 cells after treatment with a PI3K inhibitor. Magnification × 4. **H**, **I** Colony formation assay showing the colony formation number of SNB19 cells after treatment with a PI3K inhibitor. Magnification × 1. According to the mean and standard deviation *P < 0.05, **P < 0.01, ***P < 0.001, and ****P < 0.0001. ns, not significant

Through enrichment analysis, we hypothesized that FAM111B influences glioma cell behavior by modulating the PI3K/AKT pathway. For validation, we performed WB analyses to assess the levels of proteins associated with the PI3K/AKT pathway in glioma cells. The results indicated that p-PI3K and p-Akt levels were significantly reduced in sh-FAM111B cells but markedly increased in OE-FAM111B cells (Fig. 6E–G). To further determine whether the effect of FAM111B on glioma cell malignancy is mediated through the PI3K/AKT pathway, we treated the cells with a PI3K inhibitor (Wortmannin). This treatment successfully reversed the upregulatory effects of FAM111B on the PI3K/AKT pathway-associated proteins (Fig. 7A–C). Moreover, it counteracted FAM111B-induced enhancement of glioma cell proliferation, migration, and invasion (Fig. 7D–I). Collectively, these results support the hypothesis that FAM111B influences the malignant features of glioma cells primarily through the PI3K/AKT pathway, underscoring its potential as a regulatory factor in glioma pathogenesis.

### Discussion

Previous studies have indicated that FAM111B overexpression is correlated with advanced and unfavorable prognoses in various cancers, including pancreatic cancer and lung adenocarcinoma [15, 16]. For example, Sun et al. elucidated the role of FAM111B in lung adenocarcinoma regulation via the p53 pathway [15], and Wang et al. demonstrated that FAM111B suppression impeded ovarian cancer progression through the deactivation of p-AKT and augmentation of p53 expression [20]. It has been proposed that the promotion of cancer progression by FAM111B overexpression could be due to its role in the nonspecific proteolytic degradation of DNA-associated proteins, leading to compromised genome stability and altered cell cycle control [7]. However, the expression and mechanistic involvement of FAM111B in gliomas, along with its impact on malignant tumor phenotypes, remain unclear. To the best of our knowledge, this research represents the first exploration of the molecular functions of FAM111B in the context of glioma. Analysis of the TCGA_GBM&LGG and CGGA databases revealed elevated FAM111B expression in glioma tissues. Moreover, FAM111B overexpression is linked to reduced OS, PFS, and DFS, correlating with advancing disease stages. We established FAM111B as an independent prognostic marker for gliomas and a credible diagnostic indicator through both univariate and multivariate analyses. We verified the difference in FAM111B expression in glioma tissues and cell lines and constructed high- and low-expression FAM111B cell lines. CRISPR knockout edits DNA through the CRISPR-Cas9 system, thereby causing permanent changes in genes. In contrast, RNA interference (RNAi) technology uses siRNAs or shRNAs to degrade mRNA, thereby temporarily inhibiting gene expression. Therefore, CRISPR is more suitable for scenarios requiring long-term gene silencing or functional studies, whereas RNAi is more appropriate for short-term or dose-dependent experiments. Given the relatively short duration of our experiment, using RNAi technology to regulate gene expression was more appropriate. Subsequent in vitro experiments confirmed that FAM111B overexpression enhanced the oncogenic properties of glioma cells, whereas its suppression inhibited these cellular processes. Collectively, these findings highlight the pivotal role of FAM111B in glioma pathogenesis.

The PI3K/AKT pathway is fundamental in modulating diverse biological functions, including cell survival, growth, proliferation, migration, and invasion [10, 21–23]. The activation of this pathway begins when PI3K catalyzes the conversion of PIP2 to PIP3 following the activation of AKT, which is involved in numerous cellular processes [24]. The p110α subunit of PI3K, encoded by the *PIK3CA* gene, often undergoes alterations in cancer and is crucial for signal transmission from receptor tyrosine kinases to intracellular signaling cascades [25–27]. PI3K consists of a catalytic subunit, p110α, and a regulatory subunit, p85α, engaging in cell-related activities such as proliferation, growth, differentiation, migration, and survival [28]. The binding of PI3K to the membrane and its subsequent activation are facilitated by receptor tyrosine kinases through scaffold proteins or activated RAS [29, 30]. Upon activation, AKT, a serine/threonine protein kinase and downstream effector of the PI3K pathway, migrates to the membrane where it binds to PIP3 and undergoes phosphorylation and activation [31–34]. This activation process involves various downstream effectors such as GSK3, FOXO, and mTORC1, which are pivotal for cell survival, growth, and metabolism [35–37]. Notably, aberrations in the PI3K/AKT pathway can initiate numerous downstream pathways that contribute to tumorigenesis [38, 39], and the pathway’s deregulation is known to enhance glioblastoma invasion by increasing tumor cell mobility and stress resistance, thus presenting a viable target for glioblastoma treatment [40, 41]. FAM111B interacts with TACC3 in hepatocellular carcinoma. Notably, FAM111B upregulates TACC3 expression, consequently activating the PI3K/AKT signaling pathway. This activation facilitates and accelerates the malignant progression of tumors [18]. In pancreatic ductal adenocarcinoma, PRIM2 upregulates FAM111B expression, thereby enhancing RNA levels and protein stability. This interaction promotes tumor cell proliferation and migration through regulation of the PI3K/AKT pathway and epithelial-mesenchymal transition [19]. Our investigation revealed that the proteins associated with the PI3K/AKT pathway were significantly downregulated in sh-FAM11B glioblastoma cells and upregulated in OE-FAM11B cells. Treatment with a PI3K inhibitor mitigated the FAM111B’s enhancing effects of these proteins and the malignant features of glioma cells. Consequently, our results suggest that FAM111B modulates the oncogenic properties of glioma cells via the PI3K/AKT pathway.

In conclusion, our study presents novel evidence highlighting FAM111B’s significant role in glioma cell oncogenic properties. Our findings indicate that the oncogenic phenotype induced by FAM111B correlates significantly with the activation of the PI3K/AKT pathway. Consequently, FAM111B has emerged not only as a critical biomarker for the development of glioma but also as a promising novel target for therapeutic intervention in glioma treatment. Although our trial yielded promising results, it is not without limitations. Looking ahead, we recognize the importance of expanding the sample size and collaborating with multiple research centers. This will enable us to comprehensively validate both the value and broad applicability of our findings.

## Data Availability

All data and materials have been provided in the manuscript.
